# Supplementary material for: Local production of reactive oxygen species drives vincristine-induced axon degeneration
Source: Cell Death Dis. 2023 Dec 8;14(12):807. doi: 10.1038/s41419-023-06227-8 (PMC10709426; doi:10.1038/s41419-023-06227-8)
Supplement: Supplementary file 1 — Suppl Figures and legends [file 41419_2023_6227_MOESM1_ESM.pdf]

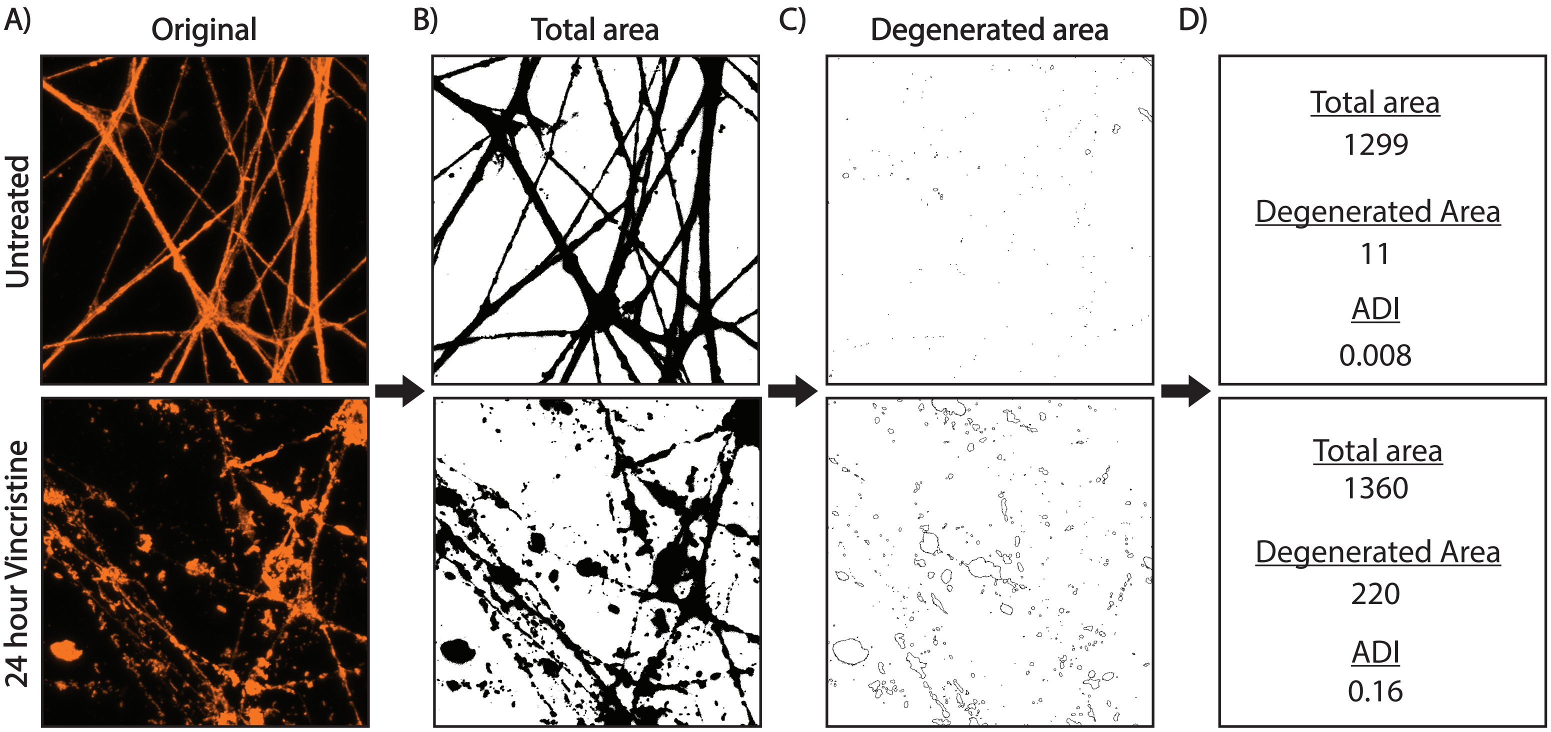
A)
Original
B)
Total area
C)
Degenerated area
D)
Untreated
24 hour Vincristine
Total area
1299
Degenerated Area
11
ADI
0.008
Total area
1360
Degenerated Area
220
ADI
0.16

Supplementary Figure 1: Quantification of axon degeneration index.

A) Representative original images of untreated axons (top) and 5 nM vincristine-treated axons (bottom).
B) Original images were binarized and adjusted to a determined threshold in FIJI that was used for all images across biological and technical replicates. Area (px^2) of the black pixels in the binarized image is considered total axonal area.
C) Binarized images are analyzed for particles with pixel units greater than or equal to 2, with a circularity of greater than or equal to 0.2. Total area of these particles is considered the degenerated area (px^2).
D) The axon degeneration index = degenerated area/ total axonal area. No differences in the total βIII tubulin staining was observed between treated and untreated conditions.

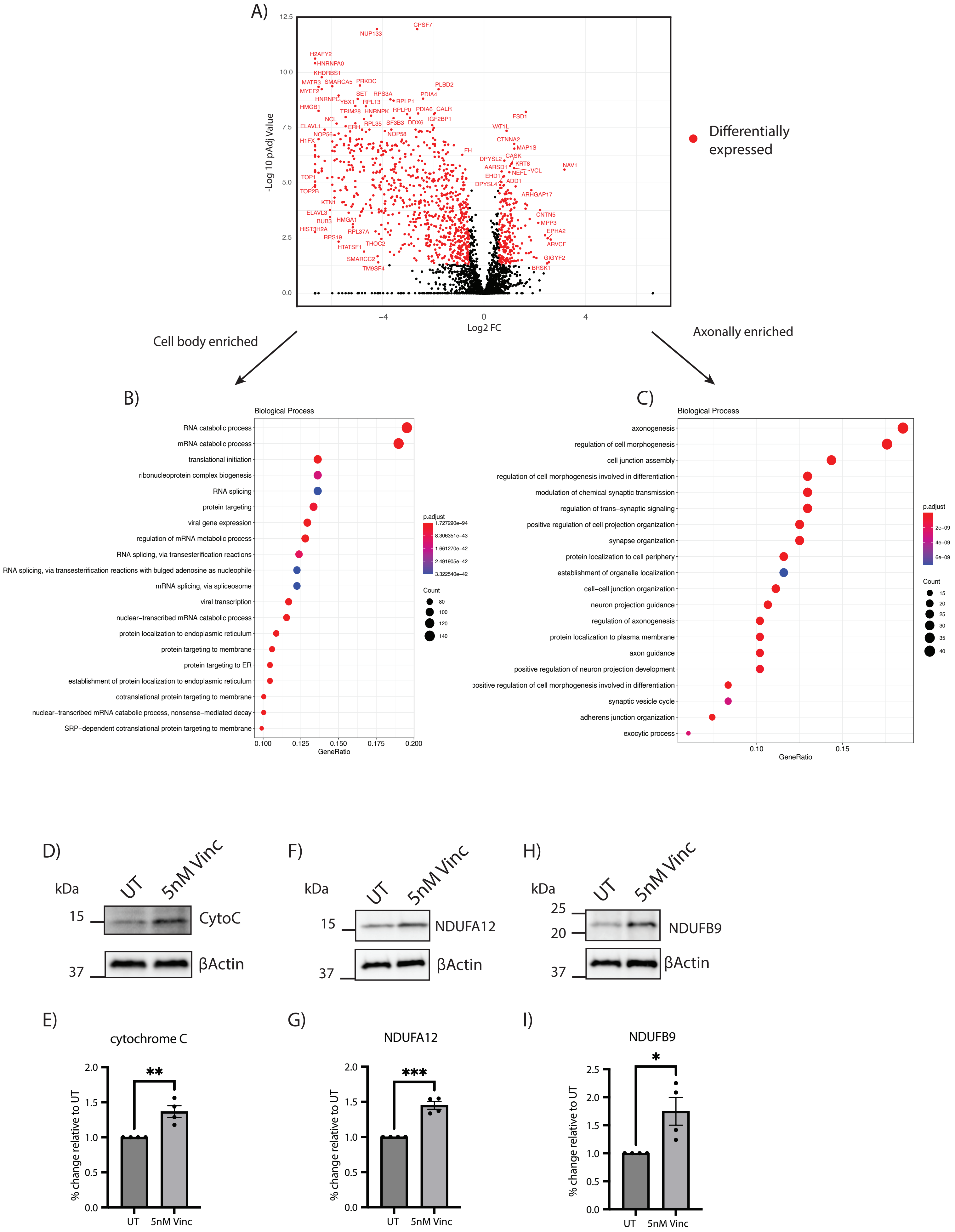

A)
Differentially expressed
-Log 10 pAdj Value
Log2 FC
CPSF7
NUP133
H2AFY2
HNRNPA0
KHDRBS1
MATR3
SMARCA5
PRKDC
MYEF2
PLBD2
SET
RPS3A
PDIA4
HNRNPC
YBX1
RPL13
RPLP1
HMGB1
TRIM28
HNRNPK
RPLP0
PDIA6
CALR
FSD1
NCL
IGF2BP1
ELAVL1
ERH
RPL35
SF3B3
DDX6
VAT1L
NOP56
NOP58
H1FX
CTNNA2
MAP1S
FH
CASK
DPYSL2
KRT8
NAV1
AARSD1
NEFL
VCL
EHD1
TOP1
ADD1
DPYSL4
TOP2B
ARHGAP17
KTN1
ELAVL3
CNTN5
BUB3
HMGA1
MPP3
HIST3H2A
EPHA2
RPL37A
RPS19
ARVCF
THOC2
HTATSF1
GIGYF2
SMARCC2
BRSK1
TM9SF4
Cell body enriched
Axonally enriched
B)
Biological Process
RNA catabolic process
mRNA catabolic process
translational initiation
ribonucleoprotein complex biogenesis
RNA splicing
protein targeting
viral gene expression
regulation of mRNA metabolic process
RNA splicing, via transesterification reactions
RNA splicing, via transesterification reactions with bulged adenosine as nucleophile
mRNA splicing, via spliceosome
viral transcription
nuclear−transcribed mRNA catabolic process
protein localization to endoplasmic reticulum
protein targeting to membrane
protein targeting to ER
establishment of protein localization to endoplasmic reticulum
cotranslational protein targeting to membrane
nuclear−transcribed mRNA catabolic process, nonsense−mediated decay
SRP−dependent cotranslational protein targeting to membrane
GeneRatio
p.adjust
Count
C)
Biological Process
axonogenesis
regulation of cell morphogenesis
cell junction assembly
regulation of cell morphogenesis involved in differentiation
modulation of chemical synaptic transmission
regulation of trans−synaptic signaling
positive regulation of cell projection organization
synapse organization
protein localization to cell periphery
establishment of organelle localization
cell−cell junction organization
neuron projection guidance
regulation of axonogenesis
protein localization to plasma membrane
axon guidance
positive regulation of neuron projection development
positive regulation of cell morphogenesis involved in differentiation
synaptic vesicle cycle
adherens junction organization
exocytic process
GeneRatio
p.adjust
Count
D)
kDa
UT
5nM Vinc
15
37
CytoC
βActin
F)
kDa
UT
5nM Vinc
15
37
NDUFA12
βActin
H)
kDa
UT
5nM Vinc
25
20
37
NDUFB9
βActin
E)
cytochrome C
**
% change relative to UT
UT
5nM Vinc
G)
NDUFA12
***
% change relative to UT
UT
5nM Vinc
I)
NDUFB9
*
% change relative to UT
UT
5nM Vinc

Supplementary Figure 2: Mass spectrometry of i^{3}Neuron cell bodies and axons reveals differences in protein composition.

A) Volcano plot showing the significant proteins up- and down-regulated in axons versus cell bodies of i^{3}Neurons. (>2 unique peptides detected, Fold Change (FC) >1.5, p. adj. value <0.05.)
B) Dot plot showing top 20 enriched biological process gene ontology analysis categories for proteins enriched in the cell body of i^{3}Neurons ranked by gene ratio.
C) Dot plot showing top 20 enriched biological process gene ontology analysis categories for proteins enriched in the axons of i^{3}Neurons ranked by gene ratio.
D) Representative western blots of i^{3}Neurons isolated axons using boyden chambers untreated (UT) and treated with 5 nM vincristine for 4 hours. Immunoblot for cytochrome C and loading control β actin. Uncropped western blots presented in Sup Fig 5.
E) Quantification of relative cytochrome C levels after 5 nM vincristine for 4 hours in the axons of i^{3}Neurons. Results normalized to UT. Results are represented as mean ± SEM. N=4 independent differentiations. Unpaired t-test. (p<0.005 **).
F) Representative western blots of i^{3}Neurons isolated axons using boyden chambers untreated (UT) and treated with 5 nM vincristine for 4 hours. Immunoblot for NDUFA12 and loading control β actin. Uncropped western blots presented in Sup Fig 5.
G) Quantification of relative NDUFA12 levels after 5 nM vincristine for 4 hours in the axons of i^{3}Neurons. Results normalized to UT. Results are represented as mean ± SEM. N=4 independent differentiations. Unpaired t-test. (p<0.001 ***).
H) Representative western blots of i^{3}Neurons isolated axons using boyden chambers untreated (UT) and treated with 5 nM vincristine for 4 hours. Immunoblot for NDUFB9 and loading control β actin. Uncropped western blots presented in Sup Fig 5.
I) Quantification of relative NDUFB9 levels after 5 nM vincristine for 4 hours in the axons of i^{3}Neurons. Results normalized to UT. Results are represented as mean ± SEM. N=4 independent differentiations. Unpaired t-test. (p<0.05 *).

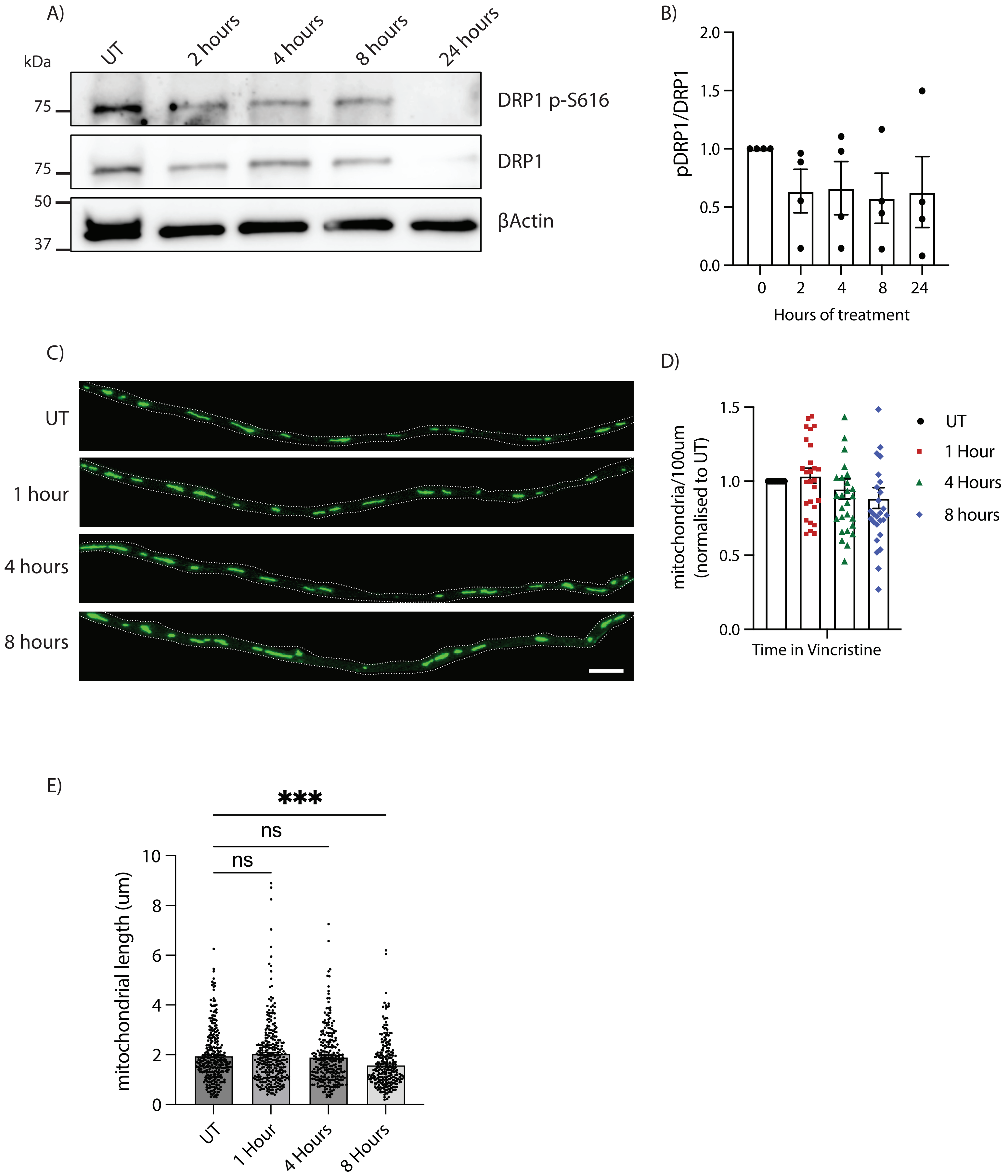
A)
kDa
UT
2 hours
4 hours
8 hours
24 hours
75
75
50
37
DRP1 p-S616
DRP1
βActin
B)
pDRP1/DRP1
2.0
1.5
1.0
0.5
0.0
0
2
4
8
24
Hours of treatment
C)
UT
1 hour
4 hours
8 hours
D)
mitochondria/100um
(normalised to UT)
1.5
1.0
0.5
0.0
Time in Vincristine
UT
1 Hour
4 Hours
8 hours
E)
***
ns
ns
mitochondrial length (um)
10
8
6
4
2
0
UT
1 Hour
4 Hours
8 Hours

Supplementary Figure 3: Vincristine does not induce mitochondrial fission in i^{3}Neurons.

A) Representative western blots of i^{3}Neurons treated with 5 nM vincristine for 2, 4, 8, and 24 hours. Immunoblot for p-S616 DRP1, total DRP1, and loading control β actin. Uncropped western blots presented in Sup Fig 5.
B) Quantification of pDRP1 (S616)/ total DRP1 levels in 5 nM i^{3}Neurons treated with 5 nM vincristine for 2, 4, 8, and 24 hours. Results normalized to UT. Results are represented as mean ± SEM. N=4 independent differentiations. One-way ANOVA. No significant changes observed.
C) Representative images of mitoGFP transduced neurons treated with 5 nM vincristine for 0, 2, 4, and 8 hours. Dotted line represents the axon. Scale bar = 10 µm.
D) Quantification of the number of axonal mitochondrial particles per 100 µm 0, 1, 4, and 8 hours after 5 nM vincristine. N=28 axons from 3 different differentiations.
E) Quantification of axon mitochondrial length 0, 1, 4 and 8 hours after 5nM vincristine. N=28 axons from 3 different differentiations. One-way ANOVA, Bonferroni correction ($p<0.001$ ***)

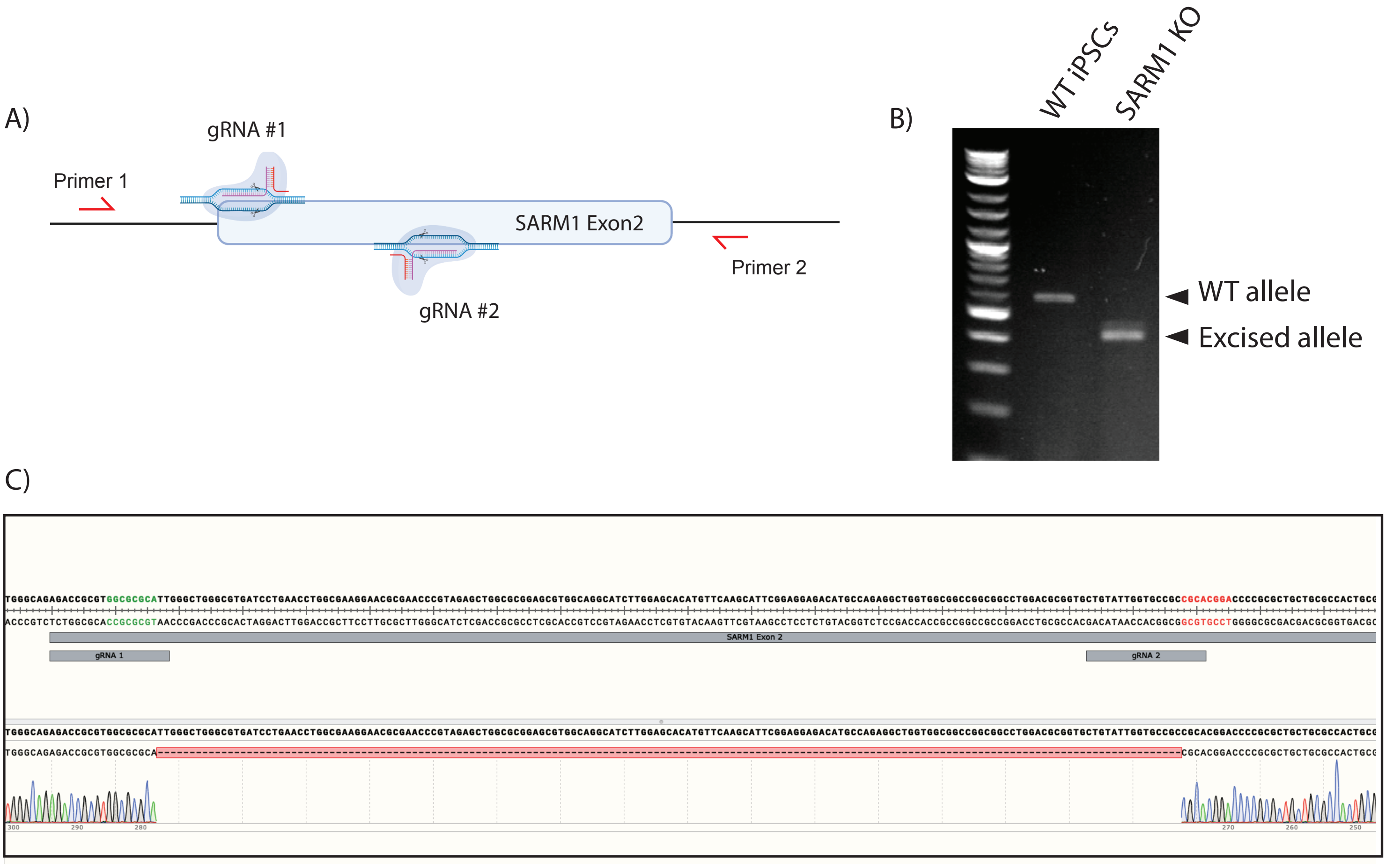
A)
gRNA #1
Primer 1
SARM1 Exon2
Primer 2
gRNA #2
B)
WT iPSCs
SARM1 KO
WT allele
Excised allele
C)
SARM1 Exon 2
gRNA 1
gRNA 2

Supplementary Figure 4: SARM1 knockout generation

A) Schematic representation of SARM1 knockout (KO) generation strategy. WT i^3 iPSCs were transfected with two gRNAs targeting *SARM1* exon 2. Primers used for knockout validation flanking *SARM1* exon 2 are shown in red.
B) PCR of WT and SARM1 KOs using KO primers. SARM1 KOs contain a 161 base pair deletion in exon 2 of the *SARM1* gene, producing a shorter gene product on the agarose gel.
C) Sanger sequencing of the *SARM1* deleted allele confirms a 161 base pair deletion resulting in a premature stop codon.
