# Supplementary figures and images for: Local production of reactive oxygen species drives vincristine-induced axon degeneration
Source: Cell Death Dis. 2023 Dec 8;14(12):807. doi: 10.1038/s41419-023-06227-8 (PMC10709426; doi:10.1038/s41419-023-06227-8)

Fig 1F:

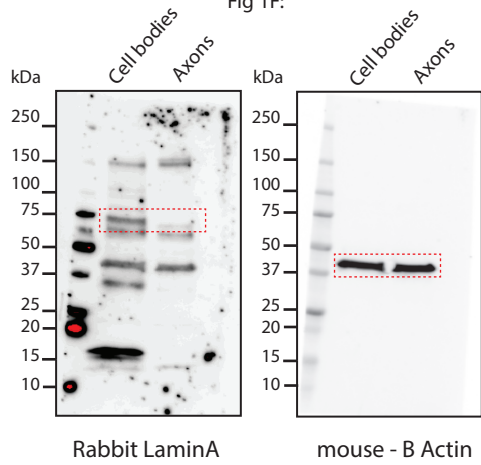

Sup Fig 2D:

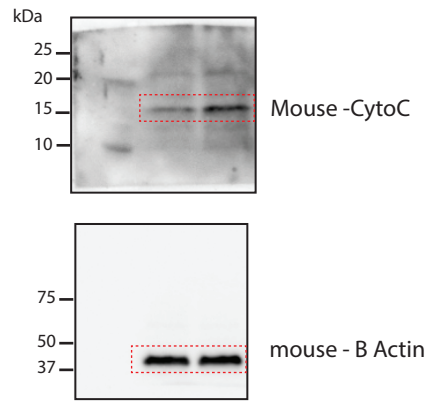

Sup Fig 2F:

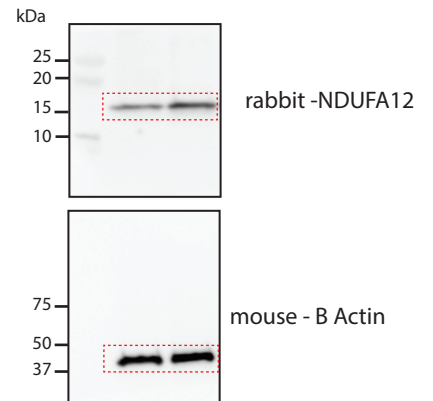

Sup Fig 3A:

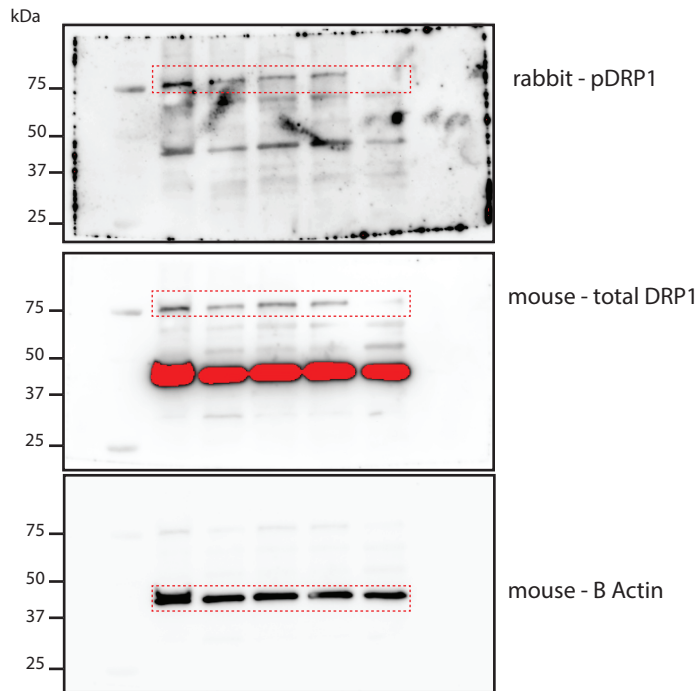

Sup Fig 2H:

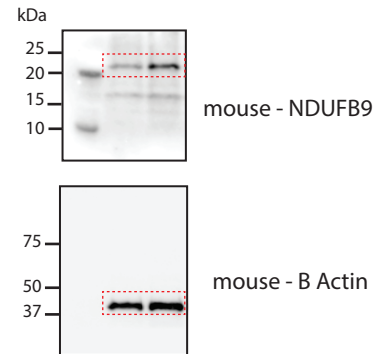

Supplement: Supplementary file 2 — Uncropped Western blots [file 41419_2023_6227_MOESM2_ESM.pdf]
